# Preparation and Properties of (YCa)(TiMn)O_3−δ_ Ceramics Interconnect of Solid Oxide Fuel Cells

**DOI:** 10.3390/ma8074239

**Published:** 2015-07-10

**Authors:** Yi-Cheng Liou, Wen-Chou Tsai, Hao-Hsuan Yen, Yung-Chia Chang

**Affiliations:** 1 Department of Electronic Engineering, Kun Shan University, No.195, Kunda Rd., Yongkang Dist., Tainan City 71070, Taiwan; E-Mails: wctsai@mail.ksu.edu.tw (W.-C.T.); s103001638@g.ksu.edu.tw (H.-H.Y.); a253591016@gmail.com (Y.-C.C.); 2 Nano Technology R&D Center, Kun Shan University, No.195, Kunda Rd., Yongkang Dist., Tainan City 71070, Taiwan

**Keywords:** (YCa)(TiMn)O_3−δ_, interconnect, solid oxide fuel cells

## Abstract

(YCa)(TiMn)O_3–δ_ ceramics prepared using a reaction-sintering process were investigated. Without any calcination involved, the mixture of raw materials was pressed and sintered directly. Y_2_Ti_2_O_7_ instead of YTiO_3_ formed when a mixture of Y_2_O_3_ and TiO_2_ with Y/Ti ratio 1/1 were sintered in air. Y_2_Ti_2_O_7_, YTiO_2.085_ and some unknown phases were detected in Y_0.6_Ca_0.4_Ti_0.6_Mn_0.4_O_3–δ_. Monophasic Y_0.6_Ca_0.4_Ti_0.4_Mn_0.6_O_3–δ_ ceramics were obtained after 1400–1500 °C sintering. Dense Y_0.6_Ca_0.4_Ti_0.4_Mn_0.6_O_3–δ_ with a density 4.69 g/cm^3^ was observed after 1500 °C/4 h sintering. Log σ for Y_0.6_Ca_0.4_Ti_0.6_Mn_0.4_O_3–δ_ increased from –3.73 Scm^–1^ at 350 °C to –2.14 Scm^–1^ at 700 °C. Log σ for Y_0.6_Ca_0.4_Ti_0.4_Mn_0.6_O_3–δ_ increased from –2.1 Scm^–1^ at 350 °C to –1.36 Scm^–1^ at 700 °C. Increasing Mn content decreased activation energy E_a_ and increased electrical conductivity. Reaction-sintering process is proved to be a simple and effective method to obtain (YCa)(TiMn)O_3–δ_ ceramics for interconnects in solid oxide fuel cells.

## 1. Introduction

Solid oxide fuel cells (SOFCs) transform chemical energy from fuels, such as natural gas, humidified hydrogen, into electrical energy with high conversion efficiency and low pollution. An SOFC includes three principal components: the electrolyte, the cathode and the anode. Each part of the SOFC needs to be compatible both physically and chemically with one another to minimize interfacial reactions. A dense electrolyte is needed to prevent gas mixing, whereas the cathode and the anode must be porous to allow gas transport to the reaction sites. SOFCs generate electricity through the oxidation of fuel at anode and the reduction of oxygen at cathode. To provide a high voltage and power output, interconnects are used to connect the cells in series.

Lanthanum chromite (LaCrO_3_) based perovskite oxides have been widely investigated as the ceramic interconnect for SOFCs due to their fairly high electrical conductivity and excellent thermodynamic stability at higher temperature [1,2]. La_0.7_Ca_0.3_Cr_0.5_Co_0.5_O_3_ was reported with very high conductivity 85 Scm^–1^ at 700 °C. However, its thermal expansion coefficient (TEC) increases from 11.12 × 10^–6^ to 19 × 10^–6^ K^–1^ due to the Co addition. This is much larger than acceptable values 11 × 10^–6^–12 × 10^–6^ K^–1^ for SOFC’s design [1,3,4,5]. Dense LaCrO_3_ based ceramics are not easy to prepare, besides, stoichiometric pellets are difficult to form because the high vapor pressure of constituent chromium at high temperature. To improve the sinterability of LaCrO_3_, some methods such as adding sintering aid, using a reducing atmosphere, and substituting lanthanum with other elements were tried by many researchers [6,7,8,9,10,11,12]. Chick *et al.* found La_0.7_Ca_x_CrO_3_ with Ca deficiency, x = 0.28 and 0.29, never attained 60% density, even at 1550 °C sintering. In contrast, the samples with Ca enrichment, x = 0.31 and 0.32, attained densities over 90% at 1400 °C sintering [12]. Mori *et al.* reported La_0.8_Sr_0.2_CrO_3_ having an average linear TEC of 9.9 × 10^–6^ K^–1^ in air and 12.2 × 10^–6^ K^–1^ in H_2_ atmosphere, in the temperature range from 50 to 1000 °C [13]. Yang and co-workers investigated La_1–x_Sr_x_CrO_3_ (x = 0–0.3) powder materials synthesized by the glycine-nitrate-process and found the maximum electrical conductivity 14.7 Scm^–1^ at 1000 °C in La_0.8_Sr_0.2_CrO_3_ sintered at 1550 °C/2 h [14]. Properties for LaCrO_3_ based oxides have been improved when part of La is substituted by Ca or Sr.

Cr-free oxides were also investigated as interconnect for SOFCs. Taguchi *et al*. reported perovskite-type (La_0.1_Ca_0.9_)(Mn_1–x_Ti_x_)O_3_ (0 ≤ x ≤ 0.9) are n-type semiconductors at low temperature. At high temperature, the manganates exhibit a metal–insulator transition in the range 0 ≤ x ≤ 0.3 [15]. Vashook *et al.* investigated perovskite-type compounds La_1–x_Ca_x_TiO_3_ (x = 0.2–1.0) and La_2(1–x)/3_Ca_x_TiO_3_ (x = 0, 0.1, 0.4 and 0.8). The crystal structures of the La_1–x_Ca_x_TiO_3_ compounds at room temperature were as orthorhombic in space group Pbnm (samples with 0.7 ≤ x ≤ 1) and as rhombohedral (x = 0.6). At room temperature three different perovskite-like structures has been found for A-deficient La_2(1–x)/3_Ca_x_TiO_3_ compound: orthorhombic Pbnm structure for x = 0.8, orthorhombic Imma structure for x = 0.4, and monoclinic P2/m (or possibly orthorhombic Cm2m) structure for x = 0.1 [16]. Kobayashi *et al*. reported the crystal structures of Ca_0.5_R_0.5_Mn_0.5_Ti_0.5_O_3_ (R = La, Nd, Eu) and Ca_0.67_R_0.33_Mn_0.33_Ti_0.67_O_3_ (R = Y, Gd, Dy, Ho, Yb) are orthorhombic Pnma (No. 62) corresponding to GdFeO_3_ type perovskite structure. The cell parameters b, c and cell volume increase with increasing ionic radius of rare earth element. The decrease in ionic radius of rare earth ions in these compounds makes increase distortion from ideal cubic perovskite [17]. Hosseini *et al*. fabricated lanthanum–manganese-doped CaTiO_3_ perovskite oxides La_0.4_Ca_0.6_Ti_1–x_Mn_x_O_3–δ_ (x = 0.0, 0.2, 0.4, 0.6) powders using an EDTA-citrate method and co-sintered as an interconnect material on an extruded porous anode substrate in a flat-tubular solid oxide fuel cell. The highest electrical conductivity occurs when x = 0.6; at 12.20 S cm^–1^ and 2.70 S cm^–1^ under oxidizing and reducing conditions [18]. Y^3+^ has typically been used to replace La^3+^ in ceramics containing lanthanum to improve their properties. We try to investigate the possibility of using (YCa)(TiMn)O_3−δ_ ceramics as interconnect for SOFCs.

In our previous studies, Pb(Mg_1/3_Nb_2/3_)O_3_ (PMN) and Pb(Fe_1/2_Nb_1/2_)O_3_ (PFN) ceramics had been prepared via a simple and effective reaction-sintering process [19,20]. Dense PMN ceramics (8.09 g/cm^3^, 99.5% of theoretic density 8.13 g/cm^3^) with maximum dielectric constant 19,900 at 1 kHz were obtained. Other Pb-based complex perovskite ceramics were also successfully produced by this reaction-sintering process. In our recent investigations, some microwave dielectric ceramics such as BaTi_4_O_9_, Ba_5_Nb_4_O_15_, Sr_5_Nb_4_O_15_, CaNb_2_O_6_, NiNb_2_O_6_, Zn_0.5_Ti_0.5_NbO_4_ and Ni_0.5_Ti_0.5_NbO_4_ were also prepared via this simple and effective reaction-sintering process [21,22,23,24,25,26]. In this study, preparation and properties of (YCa)(TiMn)O_3−δ_ ceramics via a reaction-sintering process were investigated.

## 2. Results and Discussion

**Figure 1:**
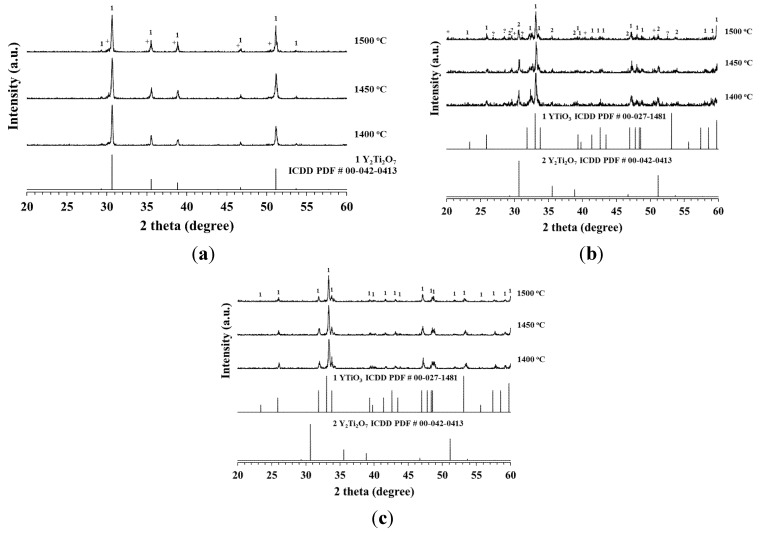
XRD patterns of the 2 h sintered (**a**) YTiO_3_ (YT); (**b**) Y_0.6_Ca_0.4_Ti_0.6_Mn_0.4_O_3−δ_(YCTM4); (**c**) Y_0.6_Ca_0.4_Ti_0.4_Mn_0.6_O_3−δ_ (YCTM6) ceramics. +: YTiO_2.085_; ?: unknown phases.

The XRD profiles for the 2 h sintered YTiO_3_ (YT), Y_0.6_Ca_0.4_Ti_0.6_Mn_0.4_O_3−δ_ (YCTM4) and Y_0.6_Ca_0.4_Ti_0.4_Mn_0.6_O_3−δ_ (YCTM6) ceramics are illustrated in Figure 1. The reflections for YT in Figure 1a match well with those of Y_2_Ti_2_O_7_ (ICDD PDF # 00-042-0413) instead those of YTiO_3_ (ICDD PDF # 00-027-1481). This implies that Y_2_Ti_2_O_7_ formed more easily than YTiO_3_ as the raw materials with Y/Ti ratio 1/1 were heated in air. Weak peaks (+) around 30° and 50.4° 2θ for YTiO_2.085_ are seen in Figure 1a. Gill *et al.* prepared Y_2_Ti_2_O_7_ from Y_2_O_3_ and Ti_2_O_3_. After 800 °C/12 h calcining, Y_2_Ti_2_O_7_ phase along with weak YTiO_2.085_ phase was detected for pellets sintered at 1500 °C/12 h. Almost monophasic YTiO_2.085_ phase was detected for pellets sintered at 1550 °C/12 h [27]. The reflections for YCTM4 in Figure 1b show that a phase with similar crystal structure of YTiO_3_ formed as the major phase and Y_2_Ti_2_O_7_ phase still formed in YCTM4. Weak peaks for YTiO_2.085_ and some unknown phases are seen in Figure 1b. The reflections for major phase are also similar to those for Y_0.33_Ca_0.67_Ti_0.67_Mn_0.33_O_3_ reported by Kobayashi *et al.* [17]. The crystal structure for YCTM4 is different from YT as Ca and Mn were added. The reflections for YCTM6 in Figure 1c show that only a phase with similar crystal structure of YTiO_3_ and Y_0.33_Ca_0.67_Ti_0.67_Mn_0.33_O_3_ reported by Kobayashi *et al.* [17] formed. Y_2_Ti_2_O_7_ phase is not detected. More Mn addition inhibited the formation of Y_2_Ti_2_O_7_, YTiO_2.085_, and unknown phases. The reaction-sintering process is proved to be a simple and effective process to obtain YT, YCTM4 and YCTM6 ceramics. The calcination step of the conventional mixed oxide route was performed during the heating up period.

**Figure 2:**
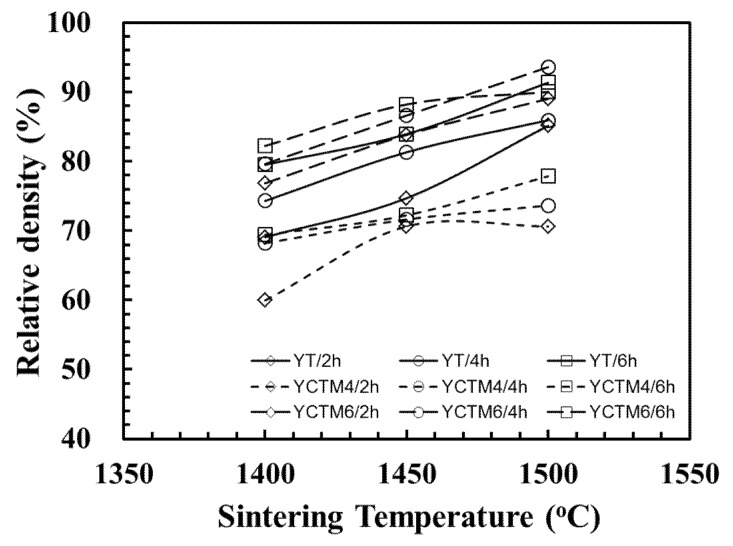
Relative density of YT, YCTM4, and YCTM6 ceramics sintered at various temperatures and soak time.

Relative density of YT, YCTM4 and YCTM6 ceramics sintered at various temperatures and soak time is shown in Figure 2. A low density, 69.1% of the theoretical density 4.98 g/cm^3^ for Y_2_Ti_2_O_7_, was found for 1400 °C/2 h sintering YT pellets. Density of YT increased with sintering temperature and soak time. Dense YT with 91.4% of the theoretical density for Y_2_Ti_2_O_7_ was observed after being sintered at 1500 °C/6 h. Gill *et al.* prepared Y_2_Ti_2_O_7_ and obtained a low density 73% of the theoretical density after 800 °C/12 h calcining and 1500 °C/12 h sintering [27]. YCTM4 pellets show lower densities. YCTM4 with 77.9% of the theoretical density ~4.97 g/cm^3^ for Y_0.6_Ca_0.4_Ti_0.6_Mn_0.4_O_3−δ_ was observed even after sintering at 1500 °C/6 h. Lower densities may be caused by the unknown phases, pores, and YTiO_2.085_ with a low density 4.28 g/cm^3^. A higher sintering temperature or a prolonged sintering period is suggested for obtaining dense YCTM4. YCTM6 pellets show higher densities. Dense YCTM6 with 93.6% of the theoretical density ~5.01 g/cm^3^ for Y_0.6_Ca_0.4_Ti_0.4_Mn_0.6_O_3−δ_ was observed after being sintered at 1500 °C/4 h. The reaction-sintering process is proved to be effective at obtaining YT and YCTM6 ceramics with a relative density higher than 90%.

**Figure 3:**
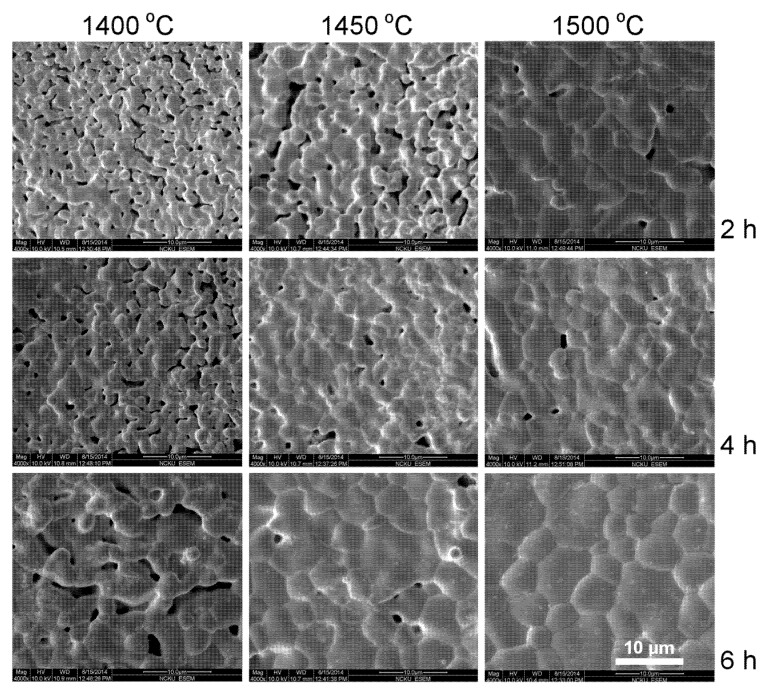
SEM photographs of the YT ceramics sintered at various temperatures and soak time.

**Figure 4:**
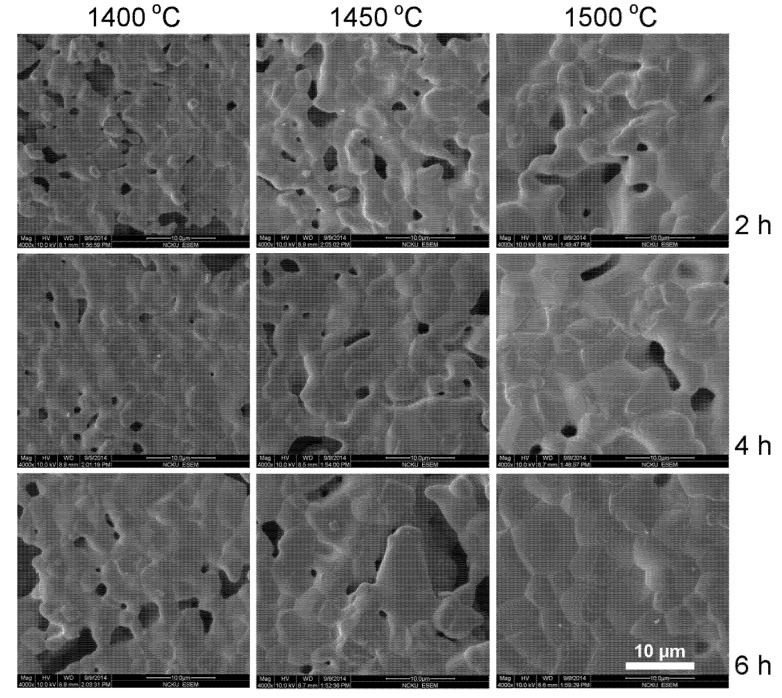
SEM photographs of the YCTM4 ceramics sintered at various temperatures and soak time.

**Figure 5:**
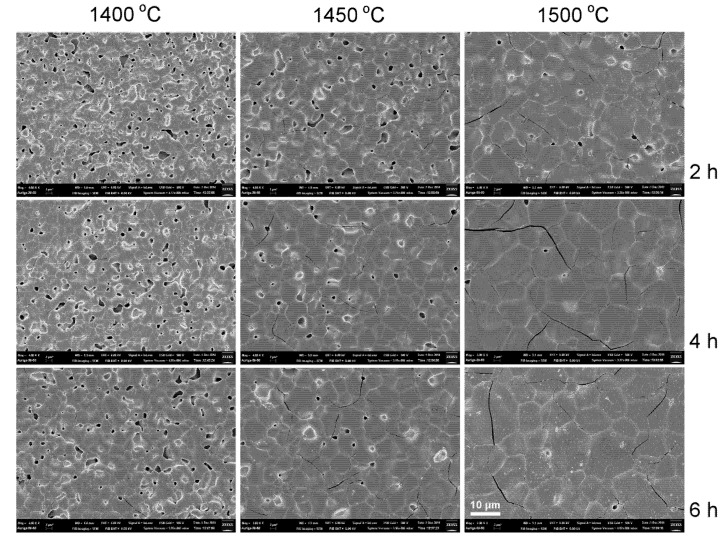
SEM photographs of the YCTM6 ceramics sintered at various temperatures and soak time.

SEM photographs of as-fired YT ceramics sintered at various temperatures and soak time are presented in Figure 3. Porous pellets with grains smaller than 3 μm are seen for 1400 °C/2 h sintering YT pellets. Grain size increased with sintering temperature and soak time. Grains > 10 μm are seen for 1500 °C/6 h sintering YT pellets. Ding *et al.* prepared Y_2_Ti_2_O_7_ with 1 mol% La_2_O_3_ and found grains smaller than 4 μm after 1450 °C/2–4 h sintering [28]. SEM photographs of the YCTM4 ceramics sintered at various temperatures and soak time are presented in Figure 4. Porous pellets with grains smaller than 8 μm are seen for 1400 °C/2 h sintering YCTM4 pellets. Grain size increased with sintering temperature and soak time. Pores disappeared and grains >10 μm are seen for 1500 °C/6 h sintering YCTM4 pellets. Grain growth increased as Ca and Mn were added into YT. Grains <4 μm are seen for La_0.4_Ca_0.6_Ti_0.6_Mn_0.4_O_3–δ_ via an EDTA-citrate method after 950 °C/5 h calcining and 1400 °C/10 h sintering [18]. Therefore, the reaction-sintering process is proved to be effective for grain growth in YT and YCTM4 ceramics. The calcinations stage and the following pulverization for the conventional solid–state reaction route could be bypassed. SEM photographs of the YCTM6 ceramics sintered at various temperatures and soak time are presented in Figure 5. Porous pellets with grains smaller than 6 μm are seen for 1400 °C/2 h sintering YCTM6 pellets. Grain size increased with sintering temperature and soak time. Pores disappeared and grains > 20 μm are seen for 1500 °C/6 h sintering YCTM6 pellets. More Mn addition increased the grain growth in YCTM6 pellets than in YCTM4 pellets. Grains <4 μm are seen for La_0.4_Ca_0.6_Ti_0.4_Mn_0.6_O_3–δ_ via an EDTA-citrate method after 950 °C/5 h calcining and 1400 °C/10 h sintering [18]. It is noted some cracks are seen in YCTM6 pellets. These cracks propagated not only along the grain boundaries but also through the grains. Besides, the amount and the size of cracks increased with sintering temperature and soak time. Sintering at temperatures below 1450 °C for a prolonged period or adding sintering aids are suggested for obtaining dense YCTM6 pellets without cracks.

**Figure 6:**
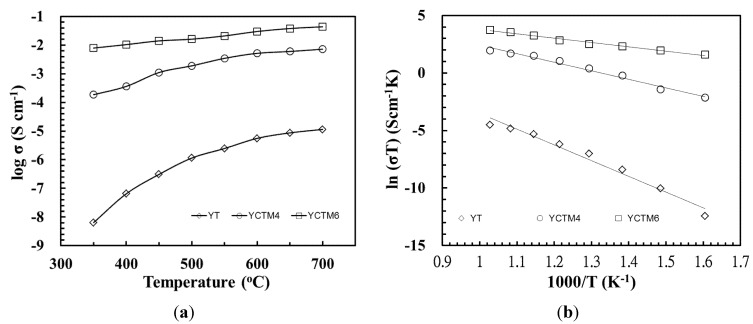
log σ and ln (σT) of 1500 °C/6 h sintering YT, YCTM4, and YCTM6 ceramics. (**a**) log σ; (**b**) ln (σT).

DC total conductivity of 1500 °C/6 h sintering YT, YCTM4, and YCTM6 ceramics are shown in Figure 6. Log σ is found from –8.19 Scm^–1^ at 350 °C to –4.94 Scm^–1^ at 700 °C for YT. Gill *et al.* prepared Y_2_Ti_2_O_7_ and obtained log σ about –7.8 Scm^–1^ at 700 °C and –6.636 Scm^–1^ at 900 °C after 800 °C/12 h calcining and 1500 °C/12 h sintering [27]. The reaction-sintering process is proved to be effective at obtaining YT ceramics with a higher conductivity even the calcination was bypassed. Log σ is found from –3.73 Scm^–1^ at 350 °C to –2.14 Scm^–1^ at 700 °C for YCTM4. Conductivity increased as Ca and Mn were added into YT. σ about 1.5 Scm^–1^ at 700 °C for La_0.4_Ca_0.6_Ti_0.6_Mn_0.4_O_3–δ_ via an EDTA-citrate method after 950 °C/5 h calcining and 1400 °C/10 h sintering was obtained [18]. Log σ is found from –2.1 Scm^–1^ at 350 °C to –1.36 Scm^–1^ at 700 °C for YCTM6. σ about 7 Scm^–1^ at 700 °C for La_0.4_Ca_0.6_Ti_0.4_Mn_0.6_O_3–δ_ via an EDTA-citrate method after 950 °C/5 h calcining and 1400 °C/10 h sintering was obtained [18]. Conductivity further increased as more Mn was added into YCTM4. A similar tendency was also observed in the study of La_0.4_Ca_0.6_Ti_1–x_Mn_x_O_3–δ_ by Hosseini *et al.* [18]. Conductivity increased as Mn content increased in La_0.4_Ca_0.6_Ti_1–x_Mn_x_O_3–δ_. According to the Arrhenius relationship [29], the experimental activation energy E_a_ can be determined from the slope of the line when natural logarithm of conductivity (ln σ) is plotted against 1/T as shown in Figure 6. E_a_ for YT, YCTM4, and YCTM6 were derived as 1.18, 0.64, and 0.33 eV, respectively. E_a_ of 1.87 eV for La_0.4_Ca_0.6_TiO_3–δ_ and E_a_ of 0.47 eV for La_0.4_Ca_0.6_Ti_0.8_Mn_0.2_O_3–δ_ was obtained via an EDTA-citrate method after 950 °C/5 h calcining and 1400 °C/10 h sintering [18]. Hosseini *et al.* thought increasing Mn content decreased E_a_ and increased electrical conductivity in air. Increasing Mn content causes the number of oxygen vacancies to increase and this in turn causes an increase in electron hole concentration [18]. A similar tendency was also observed in this study.

## 3. Experimental Section

YTiO_3_ (YT), Y_0.6_Ca_0.4_Ti_0.6_Mn_0.4_O_3−δ_ (YCTM4) and Y_0.6_Ca_0.4_Ti_0.4_Mn_0.6_O_3−δ_ (YCTM6) samples in this study were prepared from reagent-grade powders: Y_2_O_3_ (99.9%, STREM CHEMICALS, Newburyport, MA, USA), CaCO_3_ (99%, SHOWA, Tokyo, Japan), TiO_2_ (99.9%, SHOWA, Tokyo, Japan), and MnO_2_ (99.9%, J.T. Baker, Phillipsburg, NJ, USA). Appropriate amounts of raw materials for each batch were weighed and put into a PE (Polyethylene) bottle. Zirconia balls with 5 and 10 mm diameters were used for milling the raw mother powders with de-ionized water for 12 h at a speed of 500 rpm. The dried and pulverized powders were pressed into pellets with 12 mm in diameter and 1–2 mm thick. The pellets were sintered at 1400–1500 °C at a rate 10 °C/min in a covered alumina crucible in air.

The reflections of various phases for the sintered pellets were analyzed by XRD. Microstructures were analyzed by scanning electron microscopy (SEM). The density of the sintered pellets was measured using the Archimedes method. Ag electrodes were formed on both sides of the sintered pellets. Agilent 34970A Data Acquisition was used for electrical resistivity measurements at 350–700 °C.

## 4. Conclusions

(YCa)(TiMn)O_3−δ_ ceramics could be effectively obtained via a simple reaction-sintering process with the calcining stage bypassed. Y_2_Ti_2_O_7_ instead of YTiO_3_ formed when a mixture of Y_2_O_3_ and TiO_2_ with Y/Ti ratio 1/1 were heated in air. Y_2_Ti_2_O_7_, YTiO_2.085_ and some unknown phases were detected in Y_0.6_Ca_0.4_Ti_0.6_Mn_0.4_O_3-δ_. Monophasic Y_0.6_Ca_0.4_Ti_0.4_Mn_0.6_O_3−δ_ ceramics were obtained after 1400–1500 °C sintering. Y_0.6_Ca_0.4_Ti_0.6_Mn_0.4_O_3−δ_ with a density 3.87 g/cm^3^ was observed even at 1500 °C/6 h sintering. Dense Y_0.6_Ca_0.4_Ti_0.4_Mn_0.6_O_3−δ_ with a density 4.69 g/cm^3^ was observed after 1500 °C/4 h sintering. Some cracks are seen in Y_0.6_Ca_0.4_Ti_0.4_Mn_0.6_O_3−δ_ pellets. Log σ is found from –3.73 Scm^–1^ at 350 °C to –2.14 Scm^–1^ at 700 °C for Y_0.6_Ca_0.4_Ti_0.6_Mn_0.4_O_3−δ_. Log σ is found from –2.1 Scm^–1^ at 350 °C to –1.36 Scm^–1^ at 700 °C for Y_0.6_Ca_0.4_Ti_0.4_Mn_0.6_O_3−δ_. Increasing Mn content decreased activation energy E_a_ and increased electrical conductivity. Reaction-sintering process is proved to be a simple and effective method for obtaining (YCa)(TiMn)O_3−δ_ ceramics for interconnects in solid oxide fuel cells.
